# A protective-ventilation strategy reduces pulmonary complications after cardiac surgery

**DOI:** 10.1186/cc10731

**Published:** 2012-03-20

**Authors:** F Galas, A Leme, J Almeida, M Volpe, R Ianotti, J Fukushima, L Hajjar, M Amato

**Affiliations:** 1 Heart Institute, São Paulo, Brazil; 2 Federal University of Triângulo Mineiro Minas Gerais, Uberaba, Brazil; 3 Hospital das Clinicas, São Paulo, Brazil

## Introduction

Cardiac surgical procedures are associated with a high incidence of postoperative complications, increasing costs and mortality. The aim of this study is to evaluate the effect of a strategy of protective ventilation on pulmonary complications after cardiac surgery.

## Methods

We prospectively evaluated 120 patients immediately after cardiac surgery, presenting hypoxemia and PaO_2_/FiO_2 _<250. Patients were randomized to protective or conventional ventilation strategy. Protective strategy: PEEP = 13 cmH_2_O, recruitment maneuver (RM) with inspiratory pressure amplitude of 15 cmH_2_O and PEEP of 30 cmH_2_O. Conventional strategy: PEEP = 8 cmH_2_O and RM with CPAP = 20 cmH_2_O. Both patients were ventilated in pressure controlled at 6 ml/kg. Pulmonary mechanic and oxygenation parameters were collected at baseline, 15, 240 and 255 minutes after the start of treatment. Occurrence of respiratory complications was assessed in the first 5 days according to the severity score 1 to 4.

## Results

The protective group compared to the conventional group had better lung compliance (60 ± 17 vs. 48 ± 13 ml/cmH_2_O, *P *< 0.001) and higher PaO_2_/FiO_2 _(431 ± 124 vs. 229 ± 68, *P *< 0.001) at 15 minutes after the start. Also, the protective group had a lower incidence of complications after 5 days of follow-up (grade 2 = 47% vs. 55%, grade 3 = 9% vs. 13%, grade 4 = 0% vs. 3%, *P *= 0.045).

## Conclusion

A protective-ventilation strategy after cardiac surgery reduces hypoxemia, increases lung compliances and results in less respiratory complications without adverse effects.
